# Label-free *in vivo* analysis of intracellular lipid droplets in the oleaginous microalga *Monoraphidium neglectum* by coherent Raman scattering microscopy

**DOI:** 10.1038/srep35340

**Published:** 2016-10-21

**Authors:** Daniel Jaeger, Christian Pilger, Henning Hachmeister, Elina Oberländer, Robin Wördenweber, Julian Wichmann, Jan H. Mussgnug, Thomas Huser, Olaf Kruse

**Affiliations:** 1 Algae Biotechnology and Bioenergy, Faculty of Biology, Center for Biotechnology (CeBiTec), Bielefeld University, 33615 Bielefeld, Germany; 2 Biomolecular Photonics, Faculty of Physics, Bielefeld University, 33615 Bielefeld, Germany

## Abstract

Oleaginous photosynthetic microalgae hold great promise as non-food feedstocks for the sustainable production of bio-commodities. The algal lipid quality can be analysed by Raman micro-spectroscopy, and the lipid content can be imaged *in vivo* in a label-free and non-destructive manner by coherent anti-Stokes Raman scattering (CARS) microscopy. In this study, both techniques were applied to the oleaginous microalga *Monoraphidium neglectum*, a biotechnologically promising microalga resistant to commonly applied lipid staining techniques. The lipid-specific CARS signal was successfully separated from the interfering two-photon excited fluorescence of chlorophyll and for the first time, lipid droplet formation during nitrogen starvation could directly be analysed. We found that the neutral lipid content deduced from CARS image analysis strongly correlated with the neutral lipid content measured gravimetrically and furthermore, that the relative degree of unsaturation of fatty acids stored in lipid droplets remained similar. Interestingly, the lipid profile during cellular adaption to nitrogen starvation showed a two-phase characteristic with initially fatty acid recycling and subsequent *de novo* lipid synthesis. This works demonstrates the potential of quantitative CARS microscopy as a label-free lipid analysis technique for any microalgal species, which is highly relevant for future biotechnological applications and to elucidate the process of microalgal lipid accumulation.

Photosynthetic microalgae have recently gained significant attention as sustainable production hosts for a range of bio-products^1^,^2^,^3^. Algal lipid yield and relative fatty acid content are key characteristics considered when choosing an alga for biodiesel production^2^. Cellular lipids can be divided into a polar lipid fraction, mainly consisting of membrane glycerolipids, and a neutral lipid fraction, mainly consisting of triacylglycerols (TAGs)^4^. TAGs, stored as lipid droplets (LDs) within the cell, are of great interest for biodiesel production^4^,^5^. Proteome studies of isolated LDs of the model green alga *Chlamydomonas reinhardtii* have revealed a diverse set of proteins, of which more than 30 were involved in lipid metabolism^6^,^7^. The most abundant protein was termed *Major Lipid Droplet Protein* (MLDP) and was shown to act as a scaffold stabilizing LD size, and furthermore proposed to recruit other proteins to the LDs, in particular tubulin^8^.

Lipid quantification is routinely conducted by solvent extraction (for example, Folch extraction^9^), followed by fractionation and gravimetrical analysis. Subsequent HPLC or GC-MS measurements can be performed to elucidate the fatty acid composition (fatty acid profile) of the isolated lipids. Most of the established lipid extraction and characterization protocols are laborious and require a substantial amount of biomass^3^. Alternatively, lipid-specific fluorophores such as Nile Red can be employed to quantify the lipid content by fluorescence detection^10^. A powerful approach is to combine fluorophore staining with flow cytometry to rapidly monitor lipid accumulation^11^, or with fluorescent activated cell sorting to separate high-lipid mutants from a collection of transformants^12^. A major disadvantage of fluorophore staining is that many microalgal species are enclosed by a rigid cell wall, which can act as a permeability barrier for the dye^10^,^13^.

Both, lipid content and composition, can be studied by Raman micro-spectroscopy, a non-destructive and label-free method which relies on Raman scattering by probing the vibrational signature of molecules^14^. Raman spectroscopy yields information on the molecule classes inside a cell or cellular compartment, and can, for example, be used to derive the relative degree of unsaturation of fatty acids within a subcellular sample^15^. Raman scattering is also the basis for CARS, by which the distribution of a single molecular bond inside a sample can be imaged^16^,^17^. In the context of lipid analysis, aliphatic C-H_2_ bonds can be probed, which are specifically enriched in the lipid fraction, in particular in LDs. Utilizing optical microscopy with CARS as a contrast mechanism, the distribution of lipids inside the cell can thus be assessed in a label-free manner, which is a promising, cell-wall independent alternative for lipid quantification in a broad range of species. Compared to spontaneous Raman imaging with typical pixel dwell times of several seconds^18^,^19^,^20^, pixel dwell times in CARS imaging are in the range of microseconds^21^ and thus significantly faster due to a 10^5^ times higher CARS signal compared to spontaneous Raman scattering^22^. This enables the analysis of dynamic processes inside living cells without any cell preparation such as immobilization. Furthermore, the z-resolution in CARS microscopy is in the range of 650 nm compared to 2000 nm^18^ for Raman microscopy, hence the detailed lipid distribution can be imaged in 3D, especially in the case of small LDs. CARS is based on anti-Stokes scattering, a process in which a blue-shift of the photon wavelength is detected. This blue-shift is caused by the energy transfer from an excited bond vibration to a scattered photon (Stokes scattering refers to the red-shift of the signal photon wavelength that occurs while the photon interacts with the molecular bond vibration and loses energy due to the excitation of the vibrational bond). CARS microscopy employs a four-wave-mixing process in order to actively probe molecular bond vibrations of interest in a pump-probe mechanism^17^. Here, an incoming pump photon with frequency *ω*_*P*_ and a Stokes photon with frequency *ω*_*S*_ coherently excite the resonant molecular bonds collectively, while a third photon (in the special case of CARS with frequency *ω*_*P*_) probes the molecular vibration and leads to a strong anti-Stokes signal at frequency *ω*_*AS*_ = 2*ω*_*P*_ − *ω*_*S*_. Due to detecting the blue-shifted CARS signal, red-shifted background fluorescence contribution caused e.g. by the substrate does not affect the final CARS image. In contrast, spontaneous Raman scattering detects red-shifted signals, which can make it necessary to add fragile substrates such as quartz glass or magnesium-fluoride to reduce the undesired fluorescence signal.

Photosynthetic eukaryotic microalgae usually contain a large number of chlorophyll molecules, which complicates Raman analysis due to an undesired fluorescence of chlorophyll caused by two-photon excited fluorescence or direct fluorescence excitation. Although prior photobleaching reduces its contribution^19^, artefacts can potentially be introduced by this process. In order to separate the CARS signal from the undesired two-photon excited fluorescence of chlorophyll which spectrally overlaps with the CARS signal wavelengths, a very narrow band-pass filter that isolates the CARS wavelength of the C-H_2_ stretching mode and, additionally, a modulation technique can be applied^23^.

Recently, the use of CARS microscopy for lipid visualization in microalgae was demonstrated as a proof-of-principle study in the microalga *Coccomyxa subellipsoidea* C169. The analyses, however, were limited by a significant overlap between chlorophyll two-photon excited fluorescence and the CARS signals^24^. Subsequently, CARS microscopy was applied for the diatom *Phaeodactylum tricornutum*, and the CARS signal was separated from the two-photon excited chlorophyll fluorescence by time-gated detection^25^. The authors formulated a model relating their quantitative CARS data with biochemically acquired data, and a correlation between these two was observed^25^. However, *P. tricornutum* is readily stained by the lipophilic fluorophore Nile Red^26^, making the use of CARS microscopy for this microalga valuable, but not critical.

In this work, we applied CARS microscopy to the recently identified and biotechnologically promising oleaginous microalga *M. neglectum*, which is characterized by having a rigid cell wall^27^. We present an approach to separate the CARS signal from the two-photon excited chlorophyll fluorescence utilizing a modulation scheme. Lipid accumulation within intracellular LDs was induced by nitrogen starvation, and investigated by CARS microscopy. Quantification was performed in a time course analysis of LD number, size, and relative area as the respective parameters. In addition, Raman micro-spectroscopy was used in order to determine the relative degree of unsaturation of fatty acids in LDs at later stages of nitrogen starvation.

## Results

### Nile Red staining, microscopy setup and modulation approach

Staining and visualization of neutral LDs is commonly achieved with the Nile Red reagent. We applied this method to *M. neglectum*, but found that it did not work here (Supplementary Fig. S1), most likely due to obstructive properties caused by the rigid cell wall. CARS microscopy with a custom-built laser scanning microscope (Supplementary Fig. S2) was therefore established to analyse LD formation during neutral lipid accumulation. To resolve the undesired background contribution from the two-photon excited chlorophyll fluorescence originating predominately from the 816.7 nm pump beam, we amplitude-modulated the 1064 nm Stokes beam and the generated signal was demodulated by a lock-in amplifier. In this manner, the CARS signal was separated from the non-modulated fluorescence signal caused by the pump beam (Supplementary Fig. S3). As a control, the sample was illuminated by the modulated 1064 nm beam only. Here, an extremely faint signal of the chlorophyll fluorescence could still be observed (Supplementary Fig. S3). To generate the final image which contains solely lipid signals, this image (referred to as background) was subtracted from the CARS image.

### Time course of lipid accumulation in *M. neglectum*

TAG accumulation in microalgae can commonly be triggered by nitrogen starvation^4^ and can be further supported by maintaining the cells at elevated light intensities^27^,^28^,^29^. In order to investigate the time course of TAG accumulation in *M. neglectum*, we adjusted the cultures to a comparatively low cell density of ≈4 × 10^6^ cells ml^−1^ to allow optimal light penetration and subjected them to nitrogen starvation for a period of eight days. The cellular lipid content was analysed by traditional extraction protocols and the formation LDs monitored *in vivo* by CARS microscopy.

**Figure 1:**
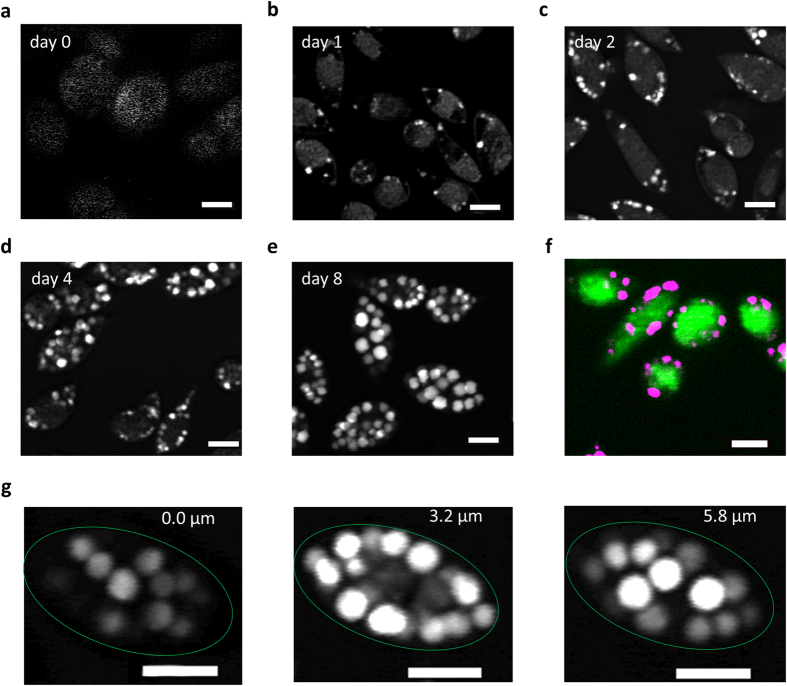
Lipid droplet formation under nitrogen starvation and localization of lipid droplets in *M. neglectum*. (**a**–**e**) Representative CARS images from day 0, 1, 2, 4, and 8 of nitrogen starvation, respectively. Bright white dots represent individual LDs. (**f**) Merged image of chlorophyll fluorescence (green) and CARS signal (magenta) at day 2, indicating the intracellular LD distribution. (**g**) Z-slices from day 8 at different heights indicate the intracellular LD distribution. Scale bar = 5 μm.

No LDs were visible under nutrient replete conditions; however, small LDs were visible after one day of nitrogen starvation (Fig. 1a,b). Subsequently, the number of LDs and their respective sizes greatly increased during the cultivation period (Fig. 1a–e), and the cells were abundantly filled with LDs after eight days (Fig. 1e,g). The LDs appeared to be restricted to the cytoplasm and to be equally and randomly distributed. The lack of a signal in the center of the cells indicated absence of LDs from the cup-shaped, central chloroplast (Fig. 1f), which was most obvious when measured at day eight (Fig. 1g).

**Figure 2:**
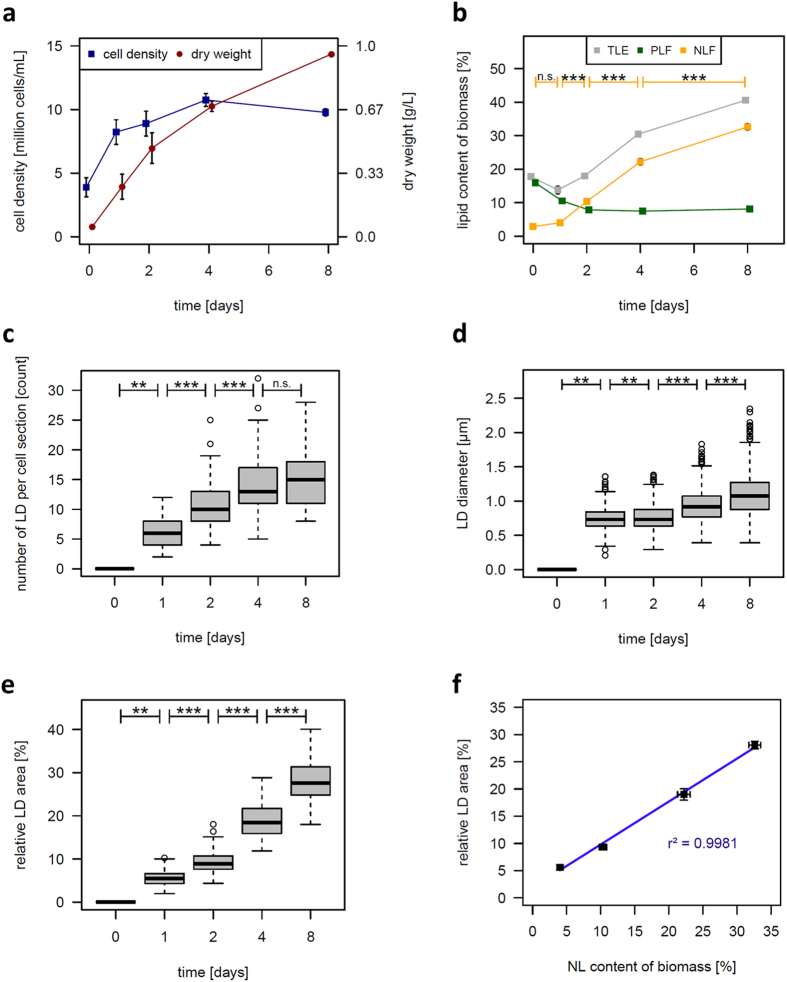
Determination of growth parameters and lipid accumulation characteristics of *M. neglectum* cell cultures during eight days of nitrogen starvation. (**a**) Cell density (blue) and dry weight (red) during the eight days of nitrogen starvation; error bars represent SEM (n = 3). (**b**) Gravimetrically determined total (TLE, total lipid extract, grey), polar (PLF, polar lipid fraction, green) and neutral (NLF, neutral lipid fraction, orange) lipid content of the biomass at the indicated time points. Error bars represent SEM (n = 3). Significance of the changes in neutral lipid content were tested by a two-sided *t*-Test. (**c**) Number of LDs from cells in a random section at different days of nitrogen starvation (n = 90 per day). (**d**) Diameter of LDs from cells in a random section at different days of nitrogen starvation (n = 0, 468, 969, 1255, and 1363 for day 0, 1, 2, 4, and 8, respectively). (**e**) Proportion of the area occupied by LDs relative to the total cell area (relative LD area, n = 90 cells per day). Box-whisker plots in (**c**–**e**): the thick lines represent the median values, the grey boxes represent the interval between the first and third quartile, the two whiskers indicate the respective 1.5x interquartile ranges, and open circles mark the outliers. Significance of changes in regard to the previous sample time point were obtained by a two-sided Wilcoxon Rank Sum Test. (**f**) Correlation of the relative LD area with the gravimetric neutral lipid content. Error bars represent SEM (n = 3). n.s. = not significant, *p < 0.05, **p < 0.01, ***p < 0.001.

Removal of nitrogen led to a cessation of cell doubling after approximately one post nutrient removal doubling, whereas, the dry weight continuously increased during the cultivation period (Fig. 2a). The total lipid amount remained constant during the first two days of nitrogen starvation, and then almost doubled after four days (Fig. 2b, grey line). To differentiate between polar and neutral lipid content of the biomass, total lipids were separated and contrasting tendencies could be observed for the polar and neutral lipid fraction. The polar lipid fraction continuously decreased during the first two days in nitrogen starvation and remained constant afterwards (Fig. 2b, green line). In contrast, the neutral lipid fraction continuously increased throughout the experiment and reached more than 30% of dry weight after eight days (Fig. 2b, orange line).

### Correlation of the gravimetric neutral lipid content with the TAG content deduced from quantitative analysis of the CARS microscopy images

In order to determine the degree of correlation between the signals detected by the microscopic CARS method and the gravimetric neutral lipid determination, the number of LDs per cell section was counted. It should be noted that this procedure can only represent an approximation of the cellular LDs, because every image represents a slice through the cell from a specific z-position (to best approximation, the longitudinal midpoint) taken at a random location. Therefore, the total number of LDs per cell can be equal or greater than the number detected in the specific section. In correspondence with the continuous increase in the gravimetrically determined neutral lipid content, the number of LDs per cell section continuously increased (Fig. 2c). The mean number of LDs per cell section increased by 150% (Fig. 2c) and the mean diameter of LDs increased by 50% (Fig. 2d). To obtain a more accurate estimation of the cellular TAG content from the CARS signal, we additionally calculated the combined LD areas, in relation to the whole cell area (relative LD area). For this purpose, the areas of all LDs (which were assumed to be of circular shapes) found in a cell section were summed and divided by the respective cell areas (assumed to be of elliptic shapes). It became apparent that the relative LD area increased fivefold (Fig. 2e), while the mean cell size remained almost constant during nitrogen starvation (Supplementary Fig. S4). The relative LD areas calculated according to the CARS signals strongly correlated with the gravimetrically determined neutral lipid contents (R^2^ = 0.99, Fig. 2f).

In a preliminary attempt, a whole cell 3D reconstruction was performed to assess the relative LD volume per cell volume (Supplementary Fig. S5). A strong correlation of the relative LD volume in the 3D model and the cellular neutral lipid content was also found (R^2^ = 0.98); however, more replicates would be required to accurately confirm this correlation. It is important to note that two separate datasets were used for the LD diameter analysis (“2D estimates”, Fig. 2d) and for the LD volume analysis (“3D estimates”, Supplementary Fig. S5), respectively. Although the results of both approaches do not perfectly align, very similar tendencies are observed. Since a far greater number of LDs were assessed for the 2D estimation (between 468–1363) compared to the 3D estimation (between 46–205), the 2D dataset can be considered to be statistically more solid. Since the amount of time necessary to acquire 3D volume estimates with the available instrument setup was far greater than the time necessary to obtain 2D estimates for the lipid content, the 3D option was not further applied.

### Raman micro-spectroscopy reveals that the ratio of fatty acid unsaturation in lipid droplets remains unchanged under prolonged nitrogen starvation

**Figure 3:**
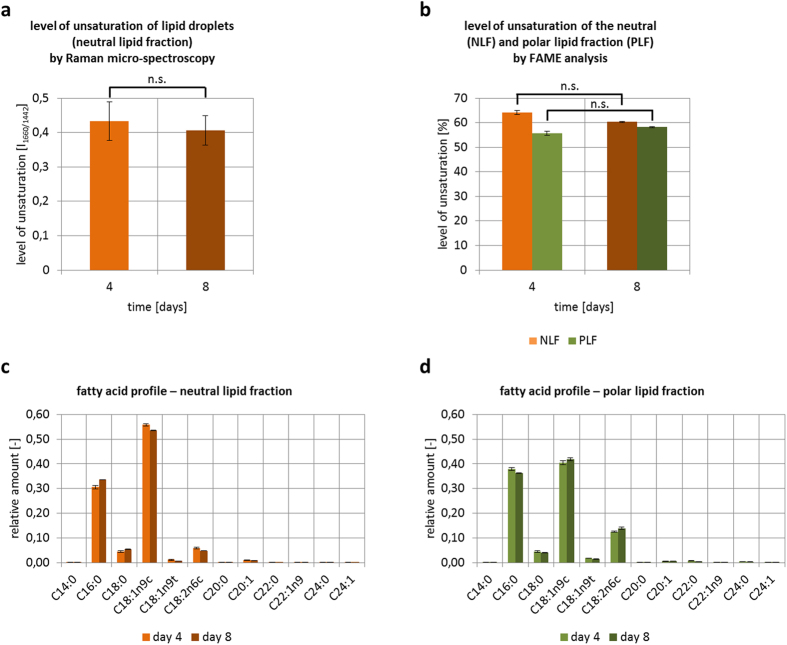
Level of unsaturation in lipid droplets and respective fatty acid composition, as determined by Raman micro-spectroscopy and concomitant biochemical analysis. (**a**) Degree of unsaturation of carbon-carbon bonds in fatty acids stored in individual LDs as determined by Raman spectroscopy. To determine the relative degree of unsaturation, 15 cells per day (5 cells per biological replicate) were quantified and the 1660 cm^−1^/1442 cm^−1^ signal ratio (1660 cm^−1^ signal indicative for C = C double bonds and 1442 cm^−1^ indicative for C-H_2_ bonds) was calculated. (**b**) Percentage of unsaturated fatty acids in the lipid profile of either the neutral lipid fraction (NLF) or the polar lipid fraction (PLF) of *M. neglectum* as determined by fatty acid methyl ester analysis (FAME) by GC-MS. (**c**,**d**) Fatty acid profile of *M. neglectum* at day 4 and day 8 from the neutral or polar lipid fraction. Significant changes in (**a**,**b**) were obtained by a two-sided Wilcoxon Rank Sum Test; n.s. = not significant. Error bars represent SEM (n = 3).

Raman micro-spectroscopy on individual LDs was performed in order to obtain further information on the chemical composition of LDs of *M. neglectum*. Day 4 and day 8 of nitrogen starvation were chosen for further analysis, because clear Raman spectra at earlier time points could not be obtained due to a low signal-to-noise ratio. The two most prominent bands in the recorded Raman spectra were attributable to carotenoids (Supplementary Fig. S6). This was also observed in another study^30^ and it should be noted that the prominent carotenoid signal is not necessarily indicative of high carotenoid abundance, because carotenoids contain delocalized π-electron systems (conjugated C = C double bonds), resulting in a resonant excitation and thus strong amplification of the Raman signal^30^. In the Raman spectra, several peaks could be assigned to lipids. We selected two peaks, the peak at 1660 cm^−1^ as an indicator for unsaturation (C = C double bond) and the peak at 1442 cm^−1^ for the overall lipid content (C-H_2_ vibration), for further analysis according to^31^. Their respective peak ratio is indicative for the relative degree of unsaturation of fatty acids stored in individual LDs. As a result, no significant differences were found between day 4 and day 8 (Fig. 3a). This was in accordance with the fatty acid profile measured for the neutral lipid fraction via fatty acid methyl ester derivatization and GC-MS (Fig. 3b–d).

## Discussion

Oleaginous microalgae represent promising production platforms for renewable lipid production in food, feed and biofuel applications. For research and development of this novel technology, it is crucial that the intracellular lipid amount and composition can be monitored in a rapid and efficient manner. Neutral lipids, such as TAGs, represent the most valuable lipid fraction for many applications. Staining of neutral lipids with the reagent Nile Red^10^ has been a commonly applied strategy in large-scale algal screening approaches such as the Aquatic Species Program^1^. A disadvantage of this technique is that it is not applicable for all oleaginous microalgal species, because cell-specific characteristics such as rigid cell walls can prevent successful dye-LD interaction. The oleaginous chlorophyte *M. neglectum* represents such a problematic species (Supplementary Fig. S1). Therefore, we used *M. neglectum* as a model to further develop label-free CARS microscopy as an alternative technique for quantitative neutral lipid monitoring.

In the present study, we applied a modulation scheme for the isolation of the CARS lipid signal, a novel approach for microalgal lipid analysis. This method employs components which are common in state-of-the-art CRS-microscopes without the need for an additional time-gated detector. Importantly, the ability of simultaneous imaging of CARS and fluorescence signals is preserved.

In *M. neglectum*, LDs could be detected and were found to increase significantly in size and number from day one to day eight of nitrogen deficient conditions (Figs 1 and 2). The diameter of most LDs was between 0.9 and 1.3 μm (Fig. 2d), corresponding to a volume of 0.5–1 μm^3^, which is far smaller than the LDs of ≥8 μm^3^ reported for the well characterized diatom species *P. tricornutum*^25^. For *C. reinhardtii*, the mean diameter of LDs stained via Nile Red was reported to be approx. 1.5 μm, which equals a volume of 1.8 μm^3^ 6. Thus, the LD size of *M. neglectum* is more similar to *C. reinhardtii* compared to *P. tricornutum* which is in accordance with the phylogenetic degree of relationship^3^. The apparent upper limit for LD size of ≤2 μm observed in *C. reinhardtii* was related to the presence of the protein MLDP^6^, in that MLDP acts as a scaffold stabilizing the LD size^8^. Since a similar upper limit for LD size also was found in *M. neglectum* (Fig. 2d), it seems likely that an analogue mechanism for LD size determination is also present in this alga. This hypothesis is strengthened by the presence of at least one homologue of the *C. reinhardtii mldp* gene in the genome of *M. neglectum* (ID = MNEG_7034).

To confirm quantitative calculations of lipid content made by CARS microscopy, the amount and composition of the intracellular lipids was simultaneously analysed biochemically with traditional solvent extraction and gravimetric determination. Both techniques strongly correlated (Fig. 2f), indicating that CARS image analysis as applied in this work allows direct quantitative calculation and reliable estimation of the TAG content. The optimal harvesting time point for maximal lipid yield of the biomass per cultivation time was four days after induction of nitrogen starvation, which is supported by both the relative LD area analysis (4.6% d^−1^) and the gravimetrically determined neutral lipid content (5.6% d^−1^).

In addition to overall lipid yield, the composition of TAGs, especially the degree of fatty acid unsaturation, is of great importance for many downstream applications. Therefore, we applied Raman micro-spectroscopy to determine whether this factor of the TAGs stored inside LDs was modified during the process of lipid accumulation. As a result, the degree of unsaturation was found to remain generally constant within the investigated period of time (Fig. 3a,b), suggesting that the cells reached a stable metabolic state of lipid synthesis under prolonged nitrogen starvation. More than 80% of the fatty acids of *M. neglectum* are either palmitic acid (C16:0) or oleic acid (C18:1) (Fig. 3c,d).

Analysis of the lipid profile indicated that *M. neglectum* cells show a two-phase reaction to prolonged nitrogen starvation: in the first phase, during early nitrogen starvation (first two days), the TAG accumulation is most likely primarily a consequence of recycling of existing polar (membrane) lipids into neutral lipids. This is indicated by the increasing neutral and simultaneously decreasing polar lipid content, while the total lipid content is unaffected (Fig. 2b). In the second phase (determined here on days 4 and 8), TAG accumulation is primarily due to *de novo* synthesis of fatty acids, which is indicated by the increasing total lipid content while the polar lipid content remains constant (Fig. 2b). Therefore, both recycling of membrane lipids^29^ and *de novo* synthesis of fatty acids^32^ likely contribute to TAG accumulation in *M. neglectum*. It should be noted that the lipid data corresponds well with values presented in a previous study where in contrast to this work, lipid profiles were obtained after five days of nitrogen starvation^27^.

In conclusion, the results of this work provide evidence that the application of label-free CARS microscopy is a valuable, non-invasive strategy to analyse the neutral lipid content in microalgae, especially in those cases where simple fluorescent dye staining cannot be applied. With this technique, it was possible for the first time to visualize the intracellular lipid content of the oleaginous green alga *M. neglectum in vivo*. Adapting CARS microscopy for high-throughput procedures would be a logical next step towards the establishment of a label-free, universally applicable technique that could enable rapid cell screenings to isolate oil-rich microalgal species or mutants. The combination of CARS with classical Raman micro-spectroscopy, allowing additional elucidation of the relative lipid composition, enables the precise determination of the optimal harvesting times for algae with specific, valuable lipid profiles.

## Methods

See Supplementary Methods for additional information.

### Cultivation conditions

Cultivations were performed in biological triplicates at room temperature (24 °C). Precultures of *Monoraphidium neglectum* (SAG 48.87) were inoculated from mixotrophic cultures (OD_750_ ≈ 0.4) and grown in Provasoli based minimal media (ProF)^33^ under autotrophic conditions (3% CO_2_ bubbling with gentle stirring under 350–400 μmol m^−2^ s^−1^ constant illumination with white light from both front and back side) for one day. On the next day, the exponentially growing precultures were washed twice with nitrogen free media (ProF –N) and adjusted to an OD_750_ of approx. 0.24 in 3 L ProF –N media. Of this, 2.5 L were transferred to 3 L vertical glass bottles (Schott, Elmsford, USA). Cultivation was performed for eight days under the conditions mentioned above. On day 1, 2, 4 and 8, 300–400 mL culture was removed for sample analysis (growth, lipid analysis and CARS microscopy).

### Lipid extractions and chromatography

Lipid extractions and chromatography were performed from 10–30 mg lyophilized biomass. After homogenization (3 × 45 s at 6,500 rpm using a Precellys 24, Peqlab, Erlangen, Germany), total lipids were extracted by modified Folch extraction^9^ and separated into polar (predominately membrane lipids) and neutral (predominately TAGs) lipid fractions according to^34^, as described previously^27^. The net total, neutral and polar lipid contents were determined gravimetrically.

### CARS image analysis

10 mL culture was concentrated 3–10 fold by gentle centrifugation and placed on ice until CARS microscopy was performed. For the setup of the custom-built CARS microscope, see Supplementary Methods. Each image was recorded three times, initially with both lasers open, then, to assess the fluorescent background signal, with either the pump beam or the Stokes beam blocked, respectively. The second and third images were subtracted from the initial CARS image by the “Image Calculator” function in ImageJ (version 1.46a, in ref. 35). The contrast was optimized for each day due to the varying CARS signal strength of the different lipid content of the cells. To approximate the number of LDs per cell, LDs in 30 cell sections per biological replicate and day (90 per day) were counted. The diameter of LDs was determined by manually fitting a straight line through a LD, and the area determined by 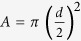 thus assuming LD as spherical objects. The sizing was carried out in ImageJ. To determine the relative LD area, 30 cells per day and replicate (90 per day) were sized. For this purpose, the cell was approximated as an ellipse and its area given by 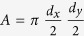, where d_x_ it the diameter of the long axis of the cell and d_y_ the diameter of the short axis of the cell. For each cell, the area of visible LDs was summed up and divided by the cell’s area, in order to obtain the relative LD area.

### Raman micro-spectroscopy

Since this analysis was not carried out on the same day of nitrogen starvation, cells were fixed with 4% paraformaldehyde. Raman spectra on the basis of spontaneous Raman spectroscopy were obtained for 5 cells per biological replicate and day (15 per day) with a custom-built Raman microscopy setup, while the laser (785 nm) is targeted at clusters of individual LDs. The peaks at 1660 cm^−1^ (C = C double bond) and at 1442 cm^−1^ (C-H_2_ vibration) were utilized to derive their ratio as an indicator of the LDs’ relative degree of unsaturation.

### Statistical analysis

Data were tested for significance (two-sided Wilcoxon Rank Sum test assuming a previous sample time point having e.g. less LDs per cell section than the current one) with a Python script incorporating R (version 2.14.1, in ref. 36) via the package “rpy2” (version 2.2.5). Significant changes of the gravimetric amounts of the neutral lipid fraction were calculated in Excel with a two-sided *t*-Test with variance set to heteroscedasticity; prior to this, technical replicates were averaged.

## Additional Information

**How to cite this article**: Jaeger, D. *et al*. Label-free *in vivo* analysis of intracellular lipid droplets in the oleaginous microalga *Monoraphidium neglectum* by coherent Raman scattering microscopy. *Sci. Rep.* **6**, 35340; doi: 10.1038/srep35340 (2016).

## Supplementary Material

Supplementary Information
