# Supplementary material for: Label-free in vivo analysis of intracellular lipid droplets in the oleaginous microalga Monoraphidium neglectum by coherent Raman scattering microscopy
Source: Sci Rep. 2016 Oct 21;6:35340. doi: 10.1038/srep35340 (PMC5073319; doi:10.1038/srep35340)
Supplement: Supplementary Information [file srep35340-s1.pdf]

Supplementary data

Manuscript title:

Label-free *in vivo* analysis of intracellular lipid droplets in the oleaginous microalga *Monoraphidium neglectum* by coherent Raman scattering microscopy

Author list:

Daniel Jaeger[1], Christian Pilger[2], Henning Hachmeister[2], Elina Oberländer[2], Robin Wörderweber[1], Julian Wichmann[1], Jan H. Mussgnug[1], Thomas Huser[2], Olaf Kruse[1]*

[1]Algae Biotechnology and Bioenergy, Center for Biotechnology, University of Bielefeld, Bielefeld 33615, Germany.

[2]Biomolecular Photonics, Department of Physics, University of Bielefeld, Bielefeld 33615, Germany.

*Address correspondence to: Olaf Kruse, olaf.kruse@uni-bielefeld.de

Present/Permanent address: Bielefeld University, Faculty of Biology, Center for Biotechnology, Universitaetsstrasse 27, 33615 Bielefeld, Germany. Phone: +49 521 106-12258, Fax: +49 521 106-12290

Supplementary Figures

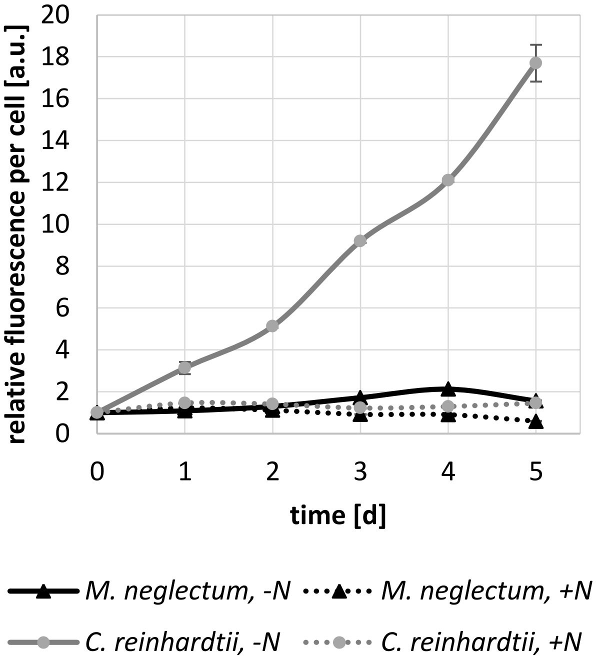

Supplementary Figure S1: Application of the Nile Red reagent for neutral lipid droplet staining. Cells from *Monoraphidium neglectum* and *Chlamydomonas reinhardtii* CC1690 (cell-wall containing strain as staining control) were transferred to nitrogen-free medium, incubated for five days to induce lipid accumulation and then subjected to the Nile Red staining procedure. The data points represent average relative fluorescence values per cell (n=4); error bars indicate SEM.

In contrast to *C. reinhardtii* (grey lines), lipid droplet formation in *M. neglectum* during the –N treatment did not result in an increase of the Nile Red fluorescence (black lines).

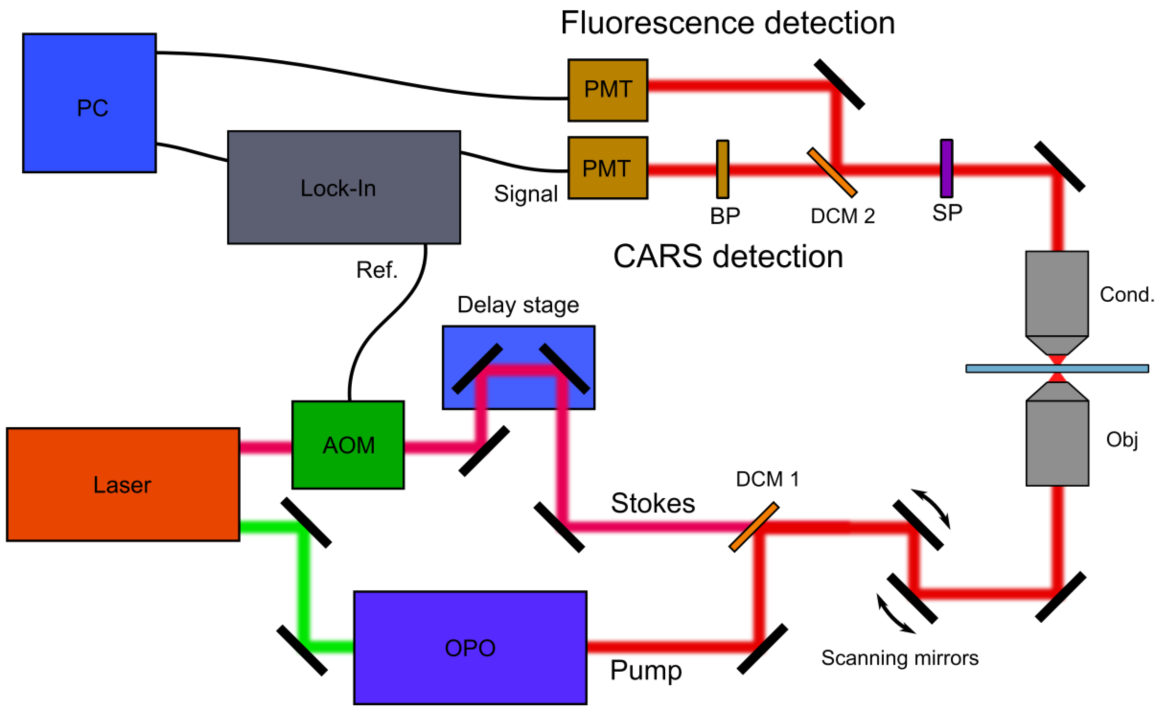

Supplementary Figure S2: Custom-built CARS microscopy setup.
The Nd:VAN laser system delivers the Stokes beam at 1064 nm and pumps the optical parametric oscillator (OPO) through frequency doubling. The OPO generates the tunable pump beam at 816.7 nm. After spatial and temporal overlapping by a dichroic mirror (DCM 1) and a delay stage, the beams are coupled into the laser scanning microscope. The CARS signal is generated using a 60x water immersion objective lens (Obj). The signals are collected by a condenser (Cond) and then separated into CARS and fluorescence contribution by a second dichroic mirror (DCM 2). A set of suitable optical filters (short-pass (SP) and band-pass (BP) filters) rejects the fundamental beams and narrows the spectral detection window for the CARS channel. In both detection arms (for fluorescence and CARS) a PMT converts the photons into electronical signals. These are acquired by an A/D converter card, which also controls the galvanometric mirrors. The MATLAB-based program ScanImage is used for the scanner control and the image formation. For the amplitude modulation an acousto-optic modulator (AOM) modulates the Stokes beam using the frequency reference (Ref.) of the lock-in amplifier, which is utilized for the demodulation of the CARS signal.

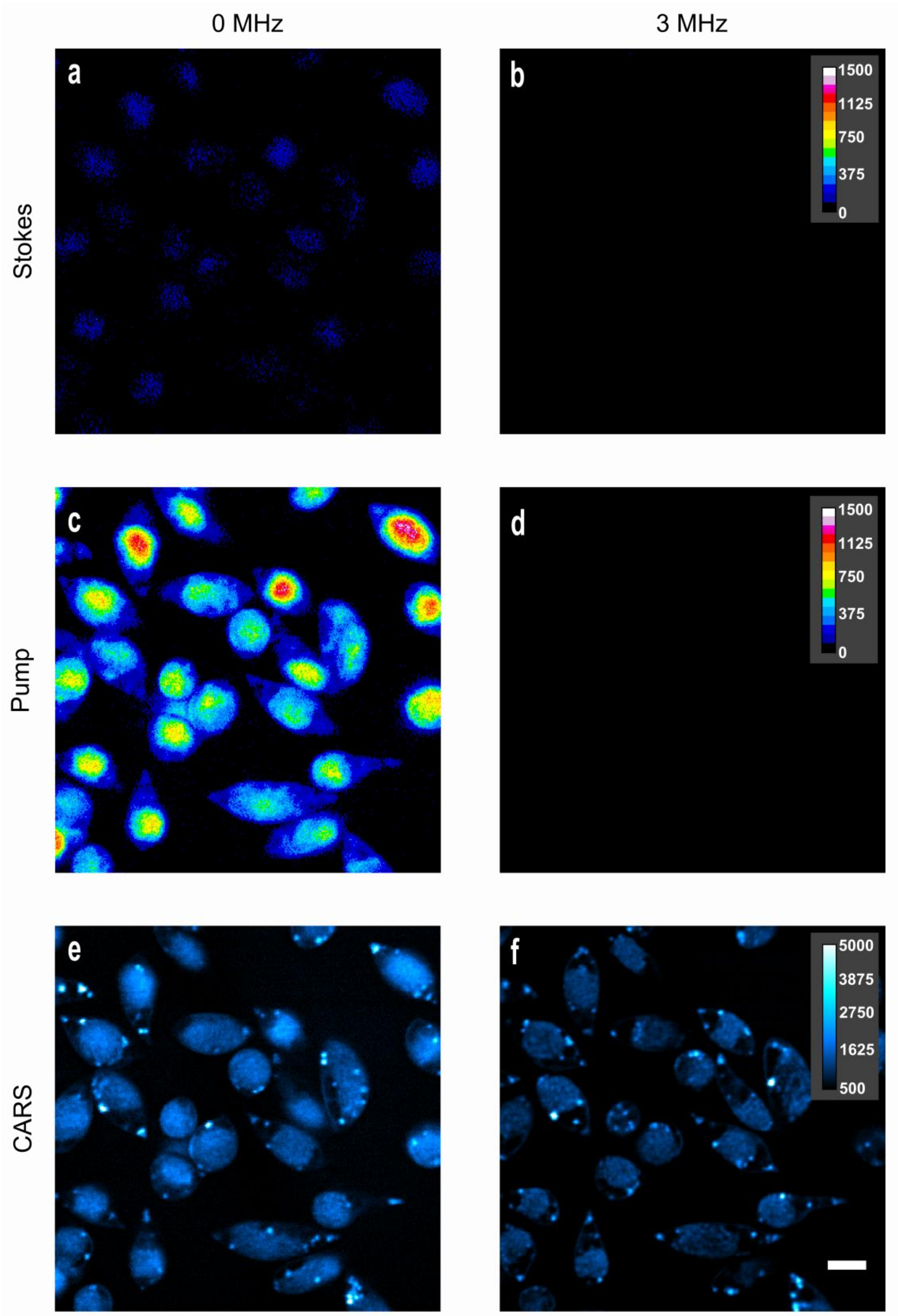

Supplementary Figure S3: Demodulation approach of the CARS signal removes two-photon excited fluorescence caused by chlorophyll.

In order to demonstrate the modulation scheme, the figures in the left column (a, c, e) display the unmodulated signal generated by the Stokes (a), pump (c) and the combination of both beams (e), respectively. The figures on the right (b, d, f) illustrate the application of the modulation scheme with a frequency of 3 MHz. Comparing figures (a) and (c) with (b) and (d) reveals that the modulation scheme efficiently filters out the two-photon fluorescence of the chlorophyll. Note that the pump beam produces a significantly higher amount of two-photon fluorescence than the Stokes beam. Figures (e) and (f) demonstrate the isolation of the CARS signal utilizing the modulation scheme. Here, the demodulated image (f) mainly consists of the resonant and non-resonant CARS signal. The figures (a) – (d) and (e) - (f) have separate lookup-tables and the scale bar indicates 5 µm. The images of *M. neglectum* microalgal cells were taken at day 1 of nitrogen starvation.

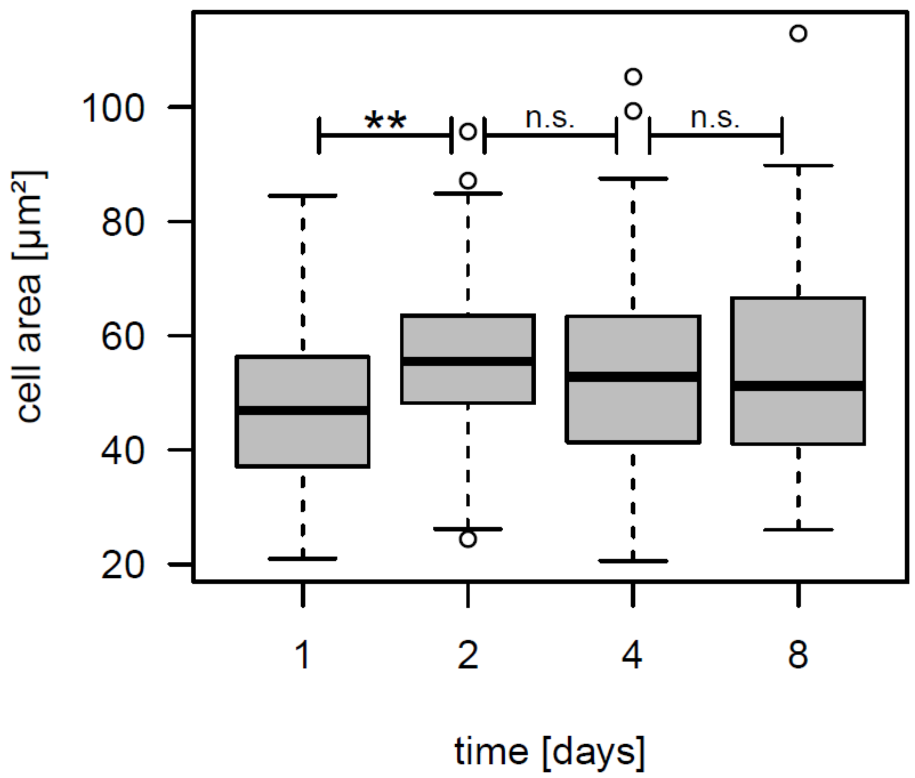

Supplementary Figure S4: Cell size of *M. neglectum* remains constant under nitrogen starvation. Box-whisker plot of the areas of 90 cells per day (30 per biological replicate). Cell area was determined by $A = \pi * \frac{d_x}{2} * \frac{d_y}{2}$, where d_x and d_y are the long and short diameter of the cell, respectively. The thick lines in the box-whisker plot represent the median values, the grey boxes represent the interval between the first and third quartile, the two whiskers indicate the respective 1.5x interquartile ranges, and open circles mark the outliers. The significance of changes with respect to the previous sample time point was tested with a two-sided Wilcoxon Rank Sum Test; n.s. = not significant; ** = $p < 0.01$.

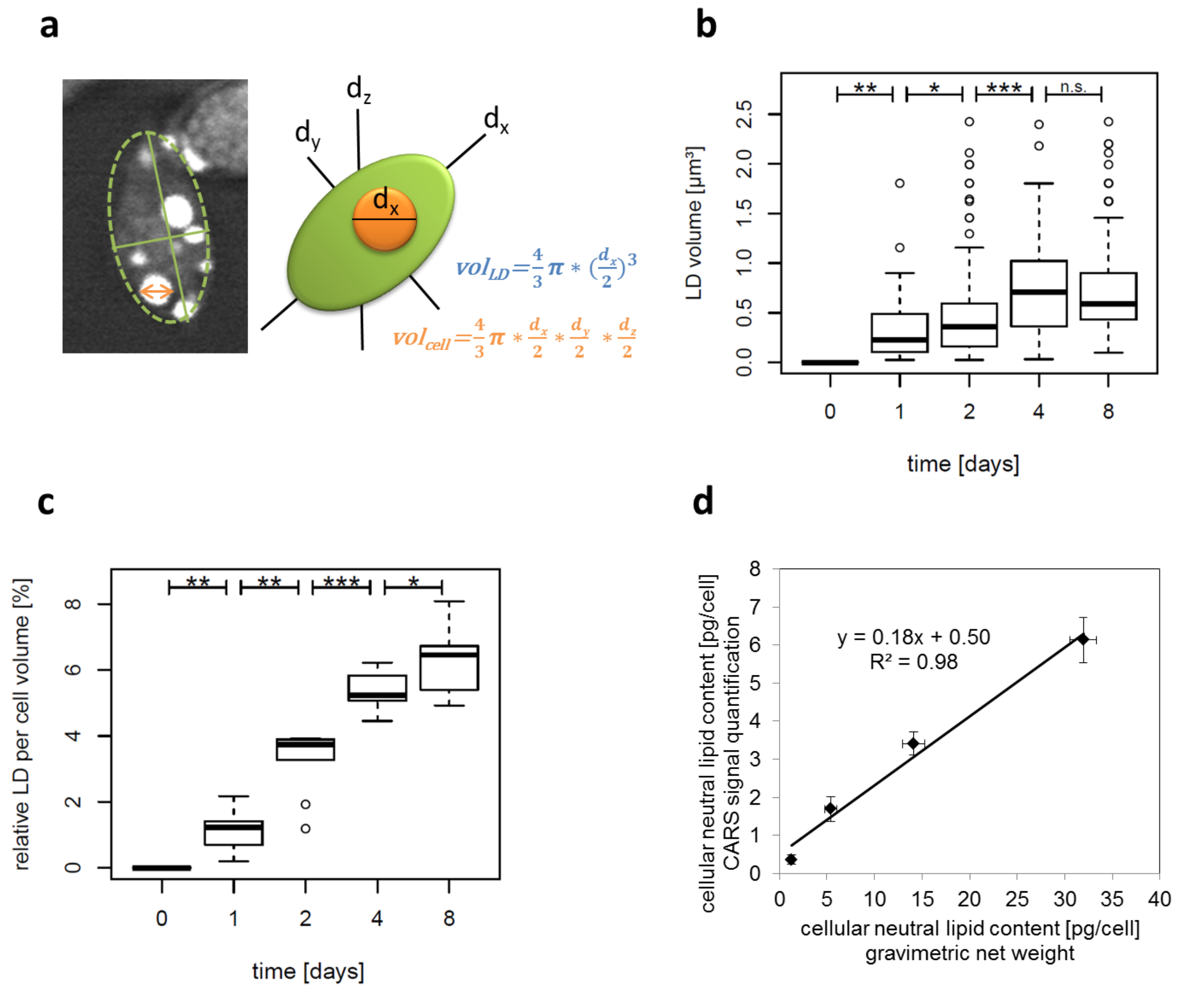

Supplementary Figure S5: Correlation of the cellular neutral lipid content determined by quantitative image analysis of 3D cell reconstructions with gravimetric solvent extraction.

(a) Outline of the quantification strategy for volumetric estimates of cells and their intracellular lipid droplets (LDs) based on 3D cell reconstructions. **(b)** Changes in LD volume during nitrogen starvation of the 3D dataset (n = 0, 46, 121, 106, and 205 for day 0, 1, 2, 4, and 8, respectively). Note that the underlying 3D-dataset (based on complete cell reconstructions) is different from the 2D dataset (based on random sections) of Figure 2d in the main article. **(c)** Volume of LDs relative to the total cell volume. For this analysis, the volumes of all LDs found in a single cell were summed up, and divided by the volume of the respective cell, in order to retrieve their relative LD volume. **(d)** Correlation of the cellular neutral lipid content determined by gravimetric analysis and the cellular neutral lipid content estimated from the relative LD volume per cell (subfigure (c), obtained by multiplying the cell weight with the relative LD volume). Error bars represent SEM (n = 3). For a description of the box-whisker plots in (b) and (c), see Supplementary Fig. S4. In (b) and (c), changes in respect to the previous sample time point were tested for significance by a two-sided Wilcoxon Rank Sum Test; n.s. = not significant, * = $p < 0.05$, ** = $p < 0.01$, *** = $p < 0.001$.

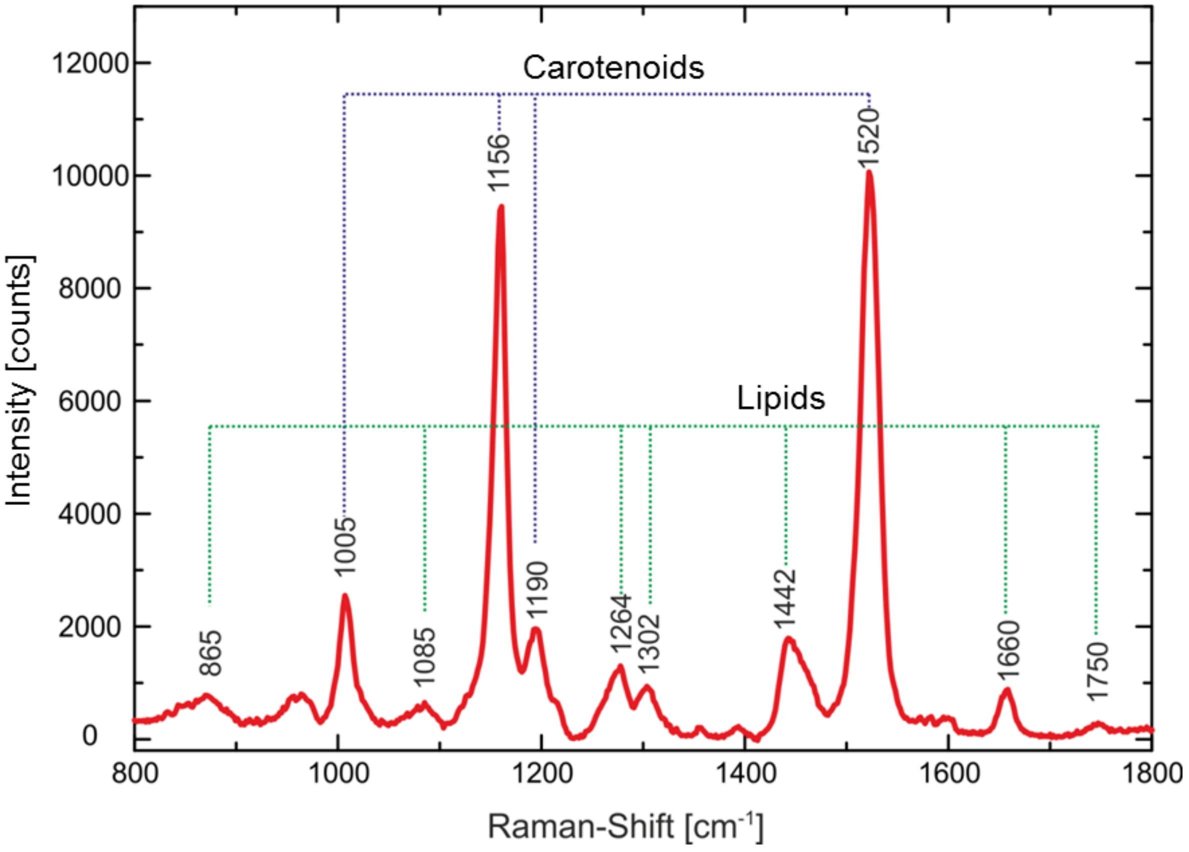

Supplementary Figure S6: Representative Raman spectrum of a lipid droplet of *M. neglectum* after eight days of nitrogen starvation.

Blue dotted lines highlight Raman peaks which are attributable to carotenoids. Note that the strong signal is not due to high carotenoid abundance, but rather the resonant excitation of delocalized π-electron systems in the pigments, leading to a strong amplification of the Raman signal. Green dotted lines indicate Raman bands which belong to lipids. The resonances at 865 cm^{-1} and 1085 cm^{-1} belong to the choline and phosphate group of lipids, respectively, indicating the presence of polar lipids in lipid droplets (LDs). Thus, it is likely that the TAG rich core of LDs is surrounded by a phospholipid layer. The signal at 1750 cm^{-1} originates from the C=O alkyl-ester vibration, indicating the presence of DAGs and TAGs inside the LD. The signal at 1302 cm^{-1} and 1442 cm^{-1} is representative of the CH_2 shear vibration of alkyl groups, whereas 1264 cm^{-1} and 1660 cm^{-1} indicates C=C stretching vibration of alkyl groups. The CH_2 signal is indicative of total lipids, since CH_2 groups belong to both saturated and unsaturated fatty acids, whereas the C=C double bond appears only in unsaturated fatty acids. The intensities of the peaks at 1264 cm^{-1} and 1302 cm^{-1} strongly depend on the structure and conformation of the fatty acid inside the lipid molecule and are as such not suited as indicators of the quantity of the respective lipid type. Therefore, the peaks at 1660 cm^{-1} and 1442 cm^{-1} were utilized to determine the amount of desaturation among the total lipids from an individual LD, which is given by the ratio of $I_{1660/1442}$. Background substraction was performed by the Lieber-algorithm [1].

Supplementary Methods

Cultivation conditions

Monoraphidium neglectum (SAG 48.87) was obtained from the Algae Collection in Göttingen. Stock cultures were maintained on plates consisting of 1.5 % Provasoli based minimal media [2] (ProF, for the composition, see [3]), supplemented with 1 % glucose (ProF + 1% glucose = PG) and 1.5 % agar. Cells were maintained mixotrophically in 50 ml PG medium in a 300 mL Erlenmeyer flask on a rotary shaker under 20 µmol m^{-2} s^{-1} constant illumination with white light from one side until stationary phase was reached (approx. one week; cultures were refreshed every week by 1:50 dilution with fresh medium). Subsequently, this culture was used for inoculation of the precultures ($OD_{750} \approx 0.4$), which were grown in minimal media (without glucose) for one day under 350 – 400 µmol m^{-2} s^{-1} constant illumination with white light from both the front and back side and 3 % CO_2 bubbling with gentle stirring. On the next day, the exponentially growing precultures were washed twice (1000 x g, 3 min) with nitrogen free minimal media (ProF –N) and adjusted to an OD_{750} of ≈0.24 in 3 L ProF –N media. 400 mL were removed for sample analysis and the rest (2.5 L) transferred to 3 L vertical glass bottles (Schott, Elmsford, USA). Cultivation was performed for 8 days under 350 – 400 µmol m^{-2} s^{-1} constant illumination with white light from both the front and back side and 3 % CO_2 bubbling with gentle stirring. At day 1, 2, 4 and 8, 400 mL culture were removed for sample analysis. All cultivations were performed in biological triplicates at room temperature (24 °C).

Cell growth parameters

To monitor cell growth, the parameters optical density (at 750 nm), cell count (Beckmann Coulter Counter) and dry biomass weight (25 mL culture washed once with distilled water and dried overnight at 105 °C) were recorded in technical triplicates per biological replicate. Technical replicates were averaged prior data analysis. Cell weight (as pg $cell^{-1}$, for subsequent determination of the cellular neutral lipid content as shown in Supplementary Fig. S5) was obtained by the following formula:

$$cell\ weight\ [pg\ cell^{-1}] = \frac{dry\ biomass\ weight\ [g\ L^{-1}]}{cell\ count\ [cells\ mL^{-1}]\ *\ 1000} * 10^{12}$$

Lipid extractions and chromatography

Lipid extractions and chromatographic analyses were performed in one (day 0 and 1) or two (day 2, 4 and 8) technical replicates per biological replicate from 10 – 30 mg lyophilized biomass. After homogenization (3 × 45 s at 6,500 rpm using a Precellys 24, Peqlab, Erlangen, Germany), total lipids were extracted and separated into polar and neutral lipids as described previously [4]. Net total,

neutral and polar lipid amounts were determined gravimetrically. The cellular neutral lipid content was obtained by:

$$cellular\ neutral\ lipid\ content\ [pg\ cell^{-1}] = \ cell\ weight\ [pg\ cell^{-1}]\ * \frac{neutral\ lipid\ content\ [\%\ of\ dry\ biomass]}{100}$$

Nile Red staining procedure (Supplementary Fig. S1)

Precultures of *C. reinhardtii* CC1690 and *M. neglectum* were inoculated to an OD_{750} of 0.05 and grown for three days. For transfer to nitrogen deficient conditions, 100 mL were washed twice with ProF –N medium (or ProF medium for the control) and resuspended in 400 mL of corresponding medium, yielding an OD_{750} of 0.2. Starvation cultures and control cultures were grown as biological duplicates in 500 mL bottles for five days with 3 % CO_2 and white light intensities of 350 – 400 µmol m^{-2} s^{-1} from both the front and back side at 24 °C.

Daily culture samples were diluted to reach final cell numbers of 1 – 3 x 10^5 cells mL^{-1}. 1 mL of diluted culture was mixed with 1 mL of deionized water containing 40 % (v v^{-1}) DMSO (final concentration = 20 % (v v^{-1})), 4 µL of Nile Red (250 µg mL^{-1} in acetone) were added and samples were vortexed for 30 s. Measurements were conducted for 200 µL of vortexed samples in transparent 96 well plates with an excitation wavelength of 530 nm and an emission wavelength of 580 nm (TECAN infinite M200 spectrofluorometer, Männedorf, Switzerland). Four technical replicates were conducted. Autofluorescence was measured by omitting addition of Nile Red and subtracted from the Nile Red fluorescence data.

CARS microscopy setup (Supplementary Fig. S2)

CARS microscopy was performed on a custom-built laser scanning microscope employing galvanometric scanning mirrors (Cambridge Technology, Galvanometer Optical Scanner, Model 6215H, Bedford, USA). An optical parametric oscillator (OPO) (Levante Emerald, APE, Berlin, Germany) is pumped by a frequency-doubled 1064 nm Nd:VAN laser (picoTRAIN, High Q laser GmbH, Rankweil, Austria) operating at 80 MHz repetition rate with pulse durations of approximately 7 ps in order to generate the appropriate wavelengths for CARS microscopy. A 60x water immersion objective lens (Olympus UPlanSApo, NA = 1.2, Olympus, Hamburg, Germany) was used to focus the spatially and temporally overlapping laser beams into the sample with a focal power of less than 35 mW to avoid destruction of the biological sample.

In order to image lipids the OPO´s wavelength was tuned to 816.7 nm as the pump beam in combination with the 1064 nm Stokes beam probing the 2845 cm^{-1} CH_2 stretching mode, which corresponds predominantly with lipids. In this case, a filter set comprised of a 950 SP, 775 SP (two times), 785 SP, 514 LP, a 660/40 band-pass filter and a tunable band-pass filter (TBP 697/13 set to 660 nm, Semrock, Rochester, USA) was utilized to isolate the CARS signal occurring at 660 nm.

All signals were detected using a photomultiplier tube (PMT) (H 9656-20 MOD *transimpedance* 2kΩ , 0-5 MHz bandwidth, Hamamatsu Photonics, Japan) by collecting the photons in forward direction through a condenser lens (U-AAC, NA = 1.4, Olympus, Hamburg, Germany). The resulting PMT signal is measured by an analogue-to-digital (A/D) converter (PCI-6110S, National Instruments, Austin, USA) and acquired by the MATLAB program ScanImage (version 3.8.1, Howard Hughes Janelia Farm Research Campus, [5]).

Due to the strong and broad two-photon fluorescence emission from chlorophyll inside the cells, an undesired background contribution from the chloroplasts was also present in the CARS lipid channel. This fluorescence was predominately excited by the pump beam. Using an acousto-optic modulator (AOM) (MT 200-A0.4-1064, Pegasus Optik, Germany) in the Stokes beam path we amplitude-modulated the 1064 nm Stokes beam intensity at a frequency of 3 MHz. Utilizing this scheme results in an overall generated signal consisting of a modulated CARS signal with a fixed frequency and a constant fluorescence signal. The combined signal measured with the PMT was then demodulated by a lock-in amplifier (HF2LI, Zurich Instruments, Switzerland) with an integration time constant of 20 µs and sent to the A/D converter card in order to isolate the CARS signal from the fluorescence.

In addition to this, images were also acquired by illuminating the sample with the modulated 1064 nm beam only. Still, a very weak signal of the chlorophyll fluorescence could be observed in the lipid channel especially at low lipid concentrations (e.g. at day 0). This image (referred to as background) was subtracted from the CARS image generating the final image, which contains only lipid signals. For additional sample information delivered by the chlorophyll fluorescence, a 600 nm long-pass filter is implemented into the signal beam path, which reflects wavelengths shorter than 600 nm. A second PMT detects the signal and generates similar to the CARS channel a fluorescence image displaying the exact position of the chloroplast in a simultaneous acquisition manner.

CARS acquisition settings

After gentle centrifugation to concentrate the culture slightly, the cells were placed on ice until CARS microscopy was performed (within one hour). 10 µL cell suspension was loaded onto a microscopic slide and incubated in the dark for 10 minutes During this incubation period, the non-motile *M. neglectum* cells settled at the bottom and due to the fast scanning times (the average acquisition time for a cell of 60 µm² (Supplementary Figure S4) was 88 ms (2D scanning mode) and 375 ms (3D scanning mode), respectively), an additional immobilization step was not required. The lateral and axial resolution of the CARS system was approximated to 400 nm and 650 nm, respectively.

For CARS microscopy the focal intensities were set to 11.6 mW for the Stokes (1064 nm) and 23.3 mW for the pump beam (816.7 nm). Here, the typical pixel dwell time was 31.25 µs with 1024 px x 1024 px per image for a field of view covering 150 µm x 150 µm to generate an overview (2D image).

In order to quantify intracellular lipids in the entire volume of the cells a 3D data set for one biological replicate per day with a step size of 500 nm in the axial (z) direction was acquired utilizing a piezo-stage (S/N 222009, Märzhäuser Wetzlar, Germany). A full 3D scan contains approx. 45 images covering the full volume of all cells (diameter 5 – 10 µm) within the field of view. In this step a smaller field view (50 µm x 50 µm) was scanned to optimize contrast and sensitivity in combination with a suitable acquisition time (approx. 30 min). These images contain 512 x 512 pixels with a pixel dwell time of 62.5 µs.

CARS image analysis

Each image was recorded three times, initially with both lasers on, then, to assess the fluorescence background, with either the pump beam or the Stokes beam blocked, respectively. The Stokes image was subtracted from the initial CARS image by the 'Image Calculator' function in ImageJ (version 1.46a). For 3D scans, only two images were recorded, the first with both lasers on and the second with the Pump beam blocked. Again, the Stokes image was subtracted from the first. These steps were automated by a homemade Python (version 2.7.3) script by writing a customized start-up macro for each image duplet or triplet and running ImageJ by the subprocess module in Python. The resulting images were then further processed manually by adding a scale bar and optimizing brightness and contrast for each day due to the varying CARS signal strength of the different lipid content of the cells by the function 'Auto' in ImageJ.

Unless a 3D scan was performed, images typically represent random sections through the cells. For the quantification of lipid droplets (LDs) per cell in these cell sections, LDs in 30 cells per biological replicate and day (90 per day) were counted. The diameter of LDs was determined by manually fitting a straight line, and the area determined by $A = \pi \left(\frac{d}{2}\right)^2$ assuming LDs as spherical objects. The cell was approximated as an ellipse and its area given by $A = \pi \frac{d_x}{2} \frac{d_y}{2}$, where d_x is the diameter of the long axis of the cell and d_y the diameter of the short axis of the cell. To determine the relative LD area, three steps were applied: first, the area of a cell was determined. Second, the areas of all LDs visible in this cell were summed up. Third, this sum of LD areas was divided by the cell area to yield the relative LD area. This procedure was repeated for 30 cells per day and replicate (90 per day).

The volume of LDs was estimated from the 3D scans. The shape of a LD was estimated as a sphere, and its volume, given by $V = \frac{4}{3}\pi \left(\frac{d}{2}\right)^3$, was calculated from the diameter of the z-section in which the LD was largest. For the relative LD volume analysis, all LDs in a single cell were quantified as well as all cells per 3D reconstruction (n = 9 per day, except day 1 where n = 8). To calculate the volume of a cell, its shape was approximated by an ellipsoid. The diameter of the height of the cell (d_z) was estimated from the number of z-sections in which the cell size remained constant, and the x and y

diameters (d_x and d_y) were determined in the z-section in which the cell was largest. The volume of this ellipsoid was calculated according to $V = \frac{4}{3}\,\pi\,\frac{d_x}{2}\,\frac{d_y}{2}\,\frac{d_z}{2}$. The sum of volumes of LDs was divided by the respective cell volume to derive the relative LD volume.

Statistical analysis

Data were collected and subsequently visualized as well as tested for significance (two-sided Wilcoxon Rank Sum test assuming a previous day having e.g. less LDs per cell section than the current one) with a Python script incorporating R (version 2.14.1) by the package 'rpy2' (version 2.2.5). Significant changes of the gravimetric neutral lipid amounts were calculated in Excel with a two-sided t-test with variance set to heteroscedasticity; prior to this, technical replicates were averaged. For the correlation analysis of the 3D relative LD volume (Supplementary Fig. S5), average values and standard deviations were obtained by creating – for each day – three lists (NL, CW, RV) with $2.5 * 10^6$ data points; those were obtained by resampling from normal distributions described by average and standard deviation of the (i) neutral lipid content (as % dry weight, list NL), (ii) cell weight (as pg $cell^{-1}$, list CW) and (iii) relative LD volume per cell (as % of cell volume, list RV). Every data point in list NL was then multiplied by its counterpart in list CW (yielding cellular neutral lipid content as pg $cell^{-1}$), and average and standard deviation were calculated for this new list, yielding the average cellular neutral lipid content and its standard deviation; this was repeated for RV and CW.

Raman spectroscopy based on spontaneous Raman scattering

Raman spectroscopy can be performed on living cells; any additional fixation is not required. Due to the fact that the measurements were not carried out in situ, cells were fixed with 4 % paraformaldehyde (PFA) in 1x phosphate-buffered saline (PBS) for 10 – 15 min and stored at 4 °C in the dark till the transportation.

Per biological replicate, 5 cells (a total of 15 cells per day) were analyzed by spontaneous Raman spectroscopy with a custom-built Raman micro-spectroscopy setup. 10 µL cell suspension in water (10^7 cells mL^{-1}) were put onto a quartz cover slip (made of quartz in order to minimize fluorescence from usual borosilicate cover slips). The clusters of individual lipid droplets were placed in the focus of a 100x oil immersion objective lens (UPLFNL, NA = 1.3, Olympus, USA) and subsequently investigated. As an excitation source a laser diode (Innovative Photonic Solutions, USA) with an output wavelength of 785 nm was used generating a focal intensity of 25 mW. The Raman spectra were detected by a spectrograph (Acton 2300i, Princeton Instruments, New Jersey, USA) and an air-cooled CCD camera (Newton DU 920P BR-DD, Andor, Belfast, UK). The exposure time was 20s. Each presented spectrum is the accumulation of three spectra which were obtained at the same sample

position. Prior to evaluation of the individual Raman bands, background subtraction was performed by the Lieber-algorithm [1].

Fatty acid profile determined by GC-MS

For GC-MS measurement, fatty acids were first converted to fatty acid methyl esters (FAMEs). For this purpose, the neutral or polar lipid extract was dissolved in 400 µL chloroform to which 0.88 mg mL^{-1} glycerol-triheptadecanoate (Sigma-Aldrich, Germany) was added as an internal standard. A blank was included to ascertain that contamination was absent. Next, 1.1 mL solution A (10 % (v v^{-1}) 37 % choric acid in methanol) was added to 100 µL dissolved lipids and incubated at 80 °C for 2 hours in sealed glass vials. After that, 1 mL of solution B (20 % (v v^{-1}) chloroform in hexane) was added and mixed for 10 min. 200 µL of the resulting upper phase were transferred to a 2 mL glass HPLC vial and 1 µL measured in an TraceGC gas chromatograph and ITQ ion trap mass spectrometer equipped with an AS 3000 autosampler (Thermo Scientific, Germany). A column (30 m x 0.25 mm VF-5 ms) coated with 0.25 µm of 5 % diphenyl and 95 % dimethylsiloxane (Varian Deutschland GmbH, Darmstadt, Germany) was used. Injection and interface temperature was set to 250 °C, ion source to 220 °C. Helium flow was 1 mL min^{-1}. The starting temperature in the oven was 80 °C for 1 min and then raised to 300 °C in steps of 6 °C min^{-1}. 20 scans s^{-1} were recorded in the mass range of 50-750 m z^{-1}. Chromatograms were analyzed with the Xcalibur software (version 2.0.7, Thermo Scientific, Germany); annotation of fatty acids was performed with the Supelco® 37 FAME mix (Sigma-Aldrich, Germany) and quantification by individual calibration curves for every FAME prior to sample analysis. The fatty acid profile was finally obtained by dividing the concentration of each individual fatty acid by the sum of concentrations of all fatty acids. All chromatograms were reviewed manually.

References

1. Lieber, C.A. and A. Mahadevan-Jansen, *Automated method for subtraction of fluorescence from biological Raman spectra.* Appl Spectrosc, 2003. **57**(11): p. 1363-7.
2. Provasoli, L., J. McLaughlin, and M. Droop, *The development of artificial media for marine algae.* Archiv für Mikrobiologie, 1957. **25**(4): p. 392-428.
3. Bogen, C., et al., *Identification of< i> Monoraphidium contortum</i> as a promising species for liquid biofuel production.* Bioresource technology, 2013. **133**: p. 622-626.
4. Bogen, C., et al., *Reconstruction of the lipid metabolism for the microalga Monoraphidium neglectum from its genome sequence reveals characteristics suitable for biofuel production.* BMC genomics, 2013. **14**(1): p. 926.
5. Pologruto, T.A., B.L. Sabatini, and K. Svoboda, *ScanImage: flexible software for operating laser scanning microscopes.* Biomed. Eng. Online, 2003. **2**(1): p. 13.
